# Therapeutic Roles of Tendon Stem/Progenitor Cells in Tendinopathy

**DOI:** 10.1155/2016/4076578

**Published:** 2016-04-19

**Authors:** Xin Zhang, Yu-cheng Lin, Yun-feng Rui, Hong-liang Xu, Hui Chen, Chen Wang, Gao-jun Teng

**Affiliations:** ^1^Department of Orthopaedics, Zhongda Hospital, School of Medicine, Southeast University, No. 87 Ding Jia Qiao, Nanjing, Jiangsu 210009, China; ^2^Department of Orthopaedics, Xishan People's Hospital, 588 Guang Rui Road, Wuxi, Jiangsu 214011, China; ^3^China Orthopedic Regenerative Medicine Group, Hangzhou, Zhejiang 310000, China; ^4^Jiangsu Key Laboratory of Molecular and Functional Imaging, Department of Radiology, Zhongda Hospital, School of Medicine, Southeast University, Nanjing, Jiangsu 210009, China

## Abstract

Tendinopathy is a tendon disorder characterized by activity-related pain, local edema, focal tenderness to palpation, and decreased strength in the affected area. Tendinopathy is prevalent in both athletes and the general population, highlighting the need to elucidate the pathogenesis of this disorder. Current treatments of tendinopathy are both conservative and symptomatic. The discovery of tendon stem/progenitor cells (TSPCs) and erroneous differentiation of TSPCs have provided new insights into the pathogenesis of tendinopathy. In this review, we firstly present the histopathological characteristics of tendinopathy and explore the cellular and molecular cues in the pathogenesis of tendinopathy. Current evidence of the depletion of the stem cell pool and altered TSPCs fate in the pathogenesis of tendinopathy has been presented. The potential regulatory factors for either tenogenic or nontenogenic differentiation of TSPCs are also summarized. The regulation of endogenous TSPCs or supplementation with exogenous TSPCs as therapeutic targets for the treatment of tendinopathy is proposed. Therefore, inhibiting the erroneous differentiation of TSPCs and regulating the differentiation of TSPCs into tendon cells might be important areas of future research and could provide new clinical treatments for tendinopathy. The current evidence suggests that TSPCs are promising therapeutic targets for the management of tendinopathy.

## 1. Introduction

With the increasing popularity of sports, an increasing number of people are beginning to take part in physical exercise. However, due to inappropriate movements, accidents, and the aging population, the morbidity of activity-related injuries, such as tendon injuries, is quickly increasing. More than 30 million tendon injuries occur worldwide each year, and the actual number is even higher because many injuries are not reported [1]. Tendinopathy is prevalent in athletes and individuals who have chronic tendon injuries or have overused their tendons [2]. Specifically, 30% of all running-related injuries and 40% of all elbow injuries in tennis players result from tendinopathy. Moreover, 32% of basketball players and 45% of volleyball players suffer from patellar tendinopathy [3].

Tendinopathy is a tendon disorder characterized by pain, swelling, tenderness, and dysfunction resulting from long-term tendon fatigue damage accumulation in the work place and from sports activities. Current treatments include eccentric exercise-based physical therapy, extracorporeal shockwave therapy, NSAIDs, corticosteroid injections, and operative management. All of these treatments are based on either theoretical rationale or limited clinical experience, rather than the specific manipulation of underlying pathophysiological pathways [4]. In fact, conservative treatments usually show short-term pain relief but lack long-term efficacy [5]. Surgery is required to remove the pathological tendon and repair tendon injuries if those conservative treatments fail. However, operative management carries a higher complication rate than other treatment options. The lack of evidence-based management of tendinopathy is due to the poor understanding of the pathogenesis of tendinopathy. Therefore, a better understanding of tendinopathy pathogenesis is essential for its effective evidence-based management.

In this review, we summarize the histopathological characteristics of tendinopathy and explore the cellular and molecular cues in the pathogenesis of tendinopathy. Current evidence of the depletion of the stem cell pool and altered tendon stem/progenitor cells (TSPCs) fate in the pathogenesis of tendinopathy has been presented. Some potential regulatory factors for either tenogenic or nontenogenic differentiation of TSPCs are also summarized. The regulation of endogenous TSPCs or supplementation with exogenous TSPCs as therapeutic targets for the treatment of tendinopathy is proposed. Therefore, inhibiting the erroneous differentiation of TSPCs and regulating the differentiation of TSPCs into tendon cells might be important areas of future research and could provide new clinical treatments for tendinopathy.

## 2. Histopathological Changes in Tendinopathy

Clinical patellar tendinopathy samples show characteristic histopathological changes, consisting of regions of hypercellularity and hypervascularity as well as regions of hypocellularity and hypovascularity and a lack of inflammatory cells [6]. Round tendon cells are separated from the pericellular matrix, and chondrogenic phenotypes are observed in unossified samples. Increased mast cell number was observed in human patellar tendinopathy compared to the early lack of inflammatory cells [7]. Ectopic bone morphogenetic protein-2 (BMP-2), BMP-4, and BMP-7 expression has been reported in clinical samples and animal models of patellar tendinopathy [6, 8]. Increased deposition and production of proteoglycan and glycosaminoglycans (GAG) suggested the disturbance of the extracellular matrix (ECM), which may be related to abnormal chondrogenesis [9]. The presence of fatty infiltration has also been noted.

All of these findings suggested that tendon healing is a failed healing or nonhealing process. Histologically, tendinopathic tissue shows a failed healing status characterized by tissue metaplasia. Tissue metaplasia including chondrocyte phenotypes, fatty infiltration, and bony deposits are observed in some patients with tendinopathy and animal model of tendinopathy [4]. The cells with repairing function failed to heal the tendon during the normal healing process, which caused tendon healing failure and the concomitant development of a series of clinical symptoms. Thus, it is possible that the functional repairing tendon cells were unable to differentiate into tenocytes but instead differentiated into osteoblasts, chondrocytes, and adipocytes under specific circumstances after tendon injury.

Therefore, it is important to determine the identity of the functional repairing tendon cells. Tendon tissue consists of tendon cells and collagen fibers, and TSPCs have recently been identified [10, 11]. The presence of chondrogenic, osteogenic, and adipogenic phenotypes in tendons suggests that the TSPCs fail to differentiate into tenocytes. Hence, we hypothesize that TSPCs are the functional repairing tendon cells.

## 3. Biological Characteristics of TSPCs

In 2007, Bi et al. identified a population of resident TSPCs in human and mouse tendons [10]. The TSPCs exhibit classical mesenchymal stem cells (MSCs) characteristics, including typical surface antigen expression, self-renewal, clonogenicity, and multidifferentiation potential. Unlike bone marrow MSCs, TSPCs express tendon-related markers in vitro, such as scleraxis and tenomodulin, and are capable of forming tendon and enthesis-like tissues when implanted in vivo.

Inspired by the evidence that multipotent stem cells existed in tendon tissue, Rui et al. successfully isolated TSPCs in the mid-substance of rat patellar tendon tissue and confirmed the clonogenicity, high proliferative potential, and osteogenic, chondrogenic, and adipogenic differentiation potentials in vitro [11]. Tan et al. further confirmed that TSPCs isolated from the mid-substance of the patellar tendon were label-retaining cells, which showed an increasing cell number and expression of proliferative, tendon-related, pluripotency, and pericyte-related markers in the window wound [12]. This study identified TSPCs in vivo. MSCs are the most studied stem cells because of their ability to differentiate into osteoblasts, chondrocytes, adipocytes, tenocytes, myotubes, neural cells, and hematopoietic-supporting stroma [13]. They are easy to isolate from bone marrow, synovium [14], skeletal muscle [15], adipose tissue [16], and cartilage [17]. However, a recent study of TSPCs showed that they exhibited higher clonogenicity, cell proliferation, and tenogenic-differentiation potential compared to bone marrow mesenchymal stem cells (BMSCs), suggesting that they could be a better cell source for musculoskeletal tissue regeneration [18]. TSPCs highly expressed CD44, CD90, CD90.1, CD90.2, CD146, Sca-1, Stro-1, Stage-Specific Embryonic Antigen-4 (SSEA-4), Oct-4, and nucleostemin, while they did not express CD18, CD31, CD34, CD45, CD106, CD117, CD144, or Flk-1 [10, 11, 19–21].  However, the expression of these surface antigens differs between young and aged TSPCs.  Zhou et al. reported that CD44 significantly increased and CD90.1 decreased in old TSPCs compared to young TSPCs [21]. Zhang and Wang found that rabbit patellar and Achilles tendon TSPCs expressed SSEA-4, Oct-4, and nucleostemin, while tenocytes did not express these markers [20]. These findings demonstrated that TSPCs occupied an idiographic cell type distinct from tenocytes.

## 4. Hypothetical Model of Altered TSPCs Fate

Recent studies reported that tendons harbored TSPCs and they could differentiate into nontenocytes [11]. Rui et al. observed that TSPCs from a collagenase-induced animal model showed increased expression of osteogenic and chondrogenic markers, decreased expression of tendon-related markers, higher cellular senescence, and lower proliferative capacity compared to TSPCs from healthy tendons [4]. Bi et al. reported that the TPSCs isolated from biglycan and fibromodulin double knockout mice model showed decreased expression of tendon markers (scleraxis and type I collagen) and higher expression of chondrocyte markers (type II collagen and aggrecan) as compared to cells isolated from wild-type mice, suggesting that the differentiation and functions of TPSCs were altered [10]. Thus, erroneous TSPCs differentiation might possibly play a role in the tendinopathy mechanism, and TSPCs might be a potential target for regulation. The successful isolation of TSPCs under optimized growth and differentiation conditions is critical for stem-cell-based tissue regenerative engineering and studies on stem cell function in tendon physiology, pathology, and disorders. A better understanding of the mechanism underlying these processes may lead to a breakthrough in the prevention and treatment of tendon injury and tendinopathy.

Tendons became more prone to tissue degeneration and injury with increased age [22]. Age-related changes have been implicated in decreased stem cell function. In several mammalian species, the expression of senescence markers, such as senescence-associated *β*-galactosidase, heterochromatin protein-1 foci, and p16^INK4A^, significantly increased with age in many tissues. Inomata et al. reported that aging or genotoxic stress induced the accumulation of DNA damage in melanocyte stem cells, resulting in the gradual depletion of the stem cell pool [23]. Kohler et al. found that aged/degenerated human TSPCs exhibited self-renewal and clonogenic deficits, while they retained their multidifferentiation potential [24]. The TSPCs pool became exhausted during tendon aging and degeneration in terms of size and functional fitness. We hypothesize that TSPC aging and degeneration and the depletion of the stem cell pool lead to failed tendon healing.

**Figure 1:**
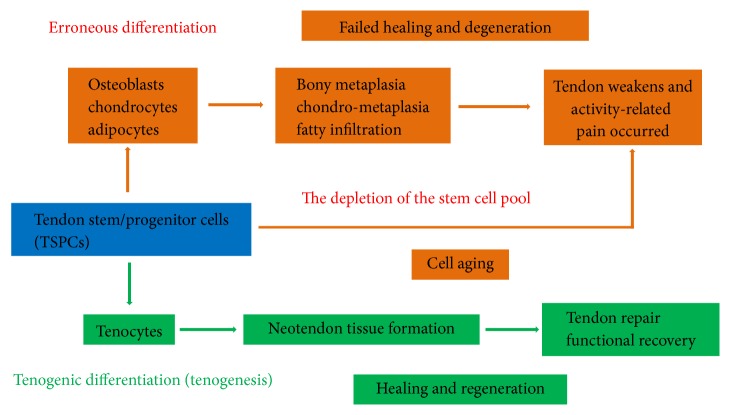
Hypothetical model of altered fate of tendon stem/progenitor cells (TSPCs) in tendinopathy and aging.

According to these recent studies, a hypothetical model has been proposed (Figure 1). Based on the model, TSPCs would differentiate into tenocytes (tenogenesis) under normal circumstances after tendon injury. However, as a result of mechanical loading and microdamage accumulation, TSPCs fail to complete the normal process and instead differentiate into chondrocytes, osteoblasts, or adipocytes. This leads to ECM degeneration and ossification. Considering the depletion of the stem cell pool during tendon aging and degeneration, sufficient healthy stem cells are essential for tendon tissue regeneration. Finally, the tendon weakens and activity-related pain occurs. Taken together, we hypothesize that TSPCs fail to differentiate into tendon fibroblasts (tenocytes) but instead differentiate into osteoblasts, chondrocytes, or adipocytes in the tendon healing process. The depletion of the stem cell pool during tendon aging and degeneration may be a possible pathogenesis of tendinopathy.

## 5. Regulatory Factors for TSPCs Differentiation

TSPCs reside in tendon tissue and are supported by a cellular microenvironment primarily composed of tenocytes and extracellular matrix. Multiple factors could regulate TSPCs differentiation, either promoting or inhibiting tenogenic differentiation. The effect of these factors, both nontenogenic differentiation factors and tenogenic differentiation factors, may be causative (Table 1).

### 5.1. Erroneous Differentiation Factors

#### 5.1.1. Mechanical Stimulation (MS)

Mechanical stimulation plays an important part in affecting the tendon extracellular microenvironment. Tenocytes do not differentiate under static conditions [50]. Repetitive tensile loading is a major cause of tendinopathy and calcifying tendinopathy. Rui et al. observed that both 4% and 8% mechanical loading increased BMP-2 expression, and BMP-2 could induce the osteogenic differentiation of TSPCs in vitro [26]. Zhang and Wang reported that lower stretching (4%) promoted tenogenic differentiation of TSPCs, whereas higher stretching (8%) inhibited tenogenic differentiation [25]. They also observed increased prostaglandin E2 (PGE2) production after treadmill running, and this increased PGE2 expression inhibited tendon stem cell differentiation into tenocytes [51]. Recent research showed that appropriate mechanical loading could be beneficial to tendons because of their potential to induce anabolic changes in tendon cells. However, while excessive mechanical loading caused anabolic changes in tendons, it also induced differentiation of TSPCs into nontenocytes [27]. The findings may lead to the development of degenerative tendinopathy frequently seen in clinical settings. A 4% dynamic mechanical stimulation increased cell proliferation, tenascin C, decorin, biglycan, and collagen type I and III (tendon-related markers) expression in TSPCs embedded in a P(LLA-CL)/Col scaffold [28]. Liu et al. reported that 8% uniaxial mechanical stress promoted osteogenic differentiation of rat TSPCs [29]. However, Chen et al. reported that human embryonic stem cells- (hESCs-) derived MSCs cultured under uniaxial static tension in vitro formed parallel collagen fibers, indicating that mechanical loading could induce hESC-MSCs into tenocytes [52]. In an in vivo study, moderate treadmill running of aging mice (9 months) resulted in the increased proliferation rate of aging TSPCs in culture, decreased lipid deposition, proteoglycan accumulation, and calcification, and increased the expression of nucleostemin in the patellar tendons [30]. This discrepancy could be due to variations in cell type, the methods used for isolating, culturing, and mechanically stimulating the cells as well as a species-related divergence.

#### 5.1.2. Alteration of Extracellular Matrix (ECM)

The ECM is the extracellular part of a multicellular structure composed of a wide range of cellular growth factors and cytokines that provide structural and biochemical support to surrounding cells. Changes to the physiological conditions of ECM after tendon injury might trigger protease activities and impair the balance between the matrix metalloproteinases (MMPs) and tissue inhibitor of metalloproteases (TIMPs) [53]. This imbalance would further induce collagen degeneration, which is critical to tendon tissue integrity. Bi et al. showed that biglycan and fibromodulin double knockout mice formed bone-like tissue instead of tendon tissue compared to wild-type mice [10]. These data indicated that the altered ECM was related to tendinopathy pathogenesis, and they verified these findings in tendinopathic patients [54]. Compared to TSPCs cultured on a plastic culture surface, the TSPCs exhibited higher proliferation potential and increased stemness when they were cultured on decellularized tendon matrix [31]. Further research is required to explore the mechanism how decellularized tendon matrix maintains tendon stem cell stemness. The ECM micro-/nanoarchitecture might also regulate tendon stem cell differentiation. TSPC culture in a random nanofibrous scaffold promoted osteogenic differentiation and ossified deposition [19]. Thus, it is important to note the role of the ECM in erroneous tendon stem cell differentiation.

#### 5.1.3. Prostaglandin E2 (PGE2)

The enzyme phospholipase A2 (PLA2) liberates arachidonic acid (AA) from cell membranes. COX-1 is a constitutive enzyme in most mammalian cells, whereas COX-2 is an inducible enzyme triggered by exposure to growth factors and inflammatory cytokines [55]. The COX-2 pathway converts AA into prostanoids, which can be subdivided into three main groups, prostaglandins (PGs), TxA2, and PGI2. Prostaglandin E synthase (PGES) synthesizes the stable prostanoid PGE2, and it can modulate inflammation through prostaglandin E receptors 1–4 [56]. Zhang and Wang found that PGE2 dose-dependently decreased cell proliferation and induced osteogenic differentiation of human TSPCs [32]. PGE2 production increased in tendons subjected to repetitive mechanical loading, and PGE2 induced the nontenogenic differentiation of TSPCs [51]. Recently, the same group found that high concentrations of PGE2 (>1 ng/mL) decreased cell proliferation and nontenogenesis of human TSPCs. However, lower PGE2 concentrations (<1 ng/mL) increased cell proliferation and the expression of SSEA-4, Stro-1, Nanog, Oct-4, collagen type I, and tenascin C [33]. Furthermore, Liu et al. showed that the PI3K-Akt signaling pathway might mediate PGE2-induced BMP-2 production and the BMP-2-induced osteogenic differentiation of rat TSPCs, providing a potential mechanism of calcified deposit formation in tendinopathy [57]. Taking adipogenesis into account, insulin-like growth factor-1 (IGF-1) alone is unable to induce adipogenic differentiation of TSPCs; however, IGF-1 together with BMP-2 could significantly induce adipogenesis in TSPCs. PGE2 stimulation induced IGF-1 upregulation through the cAMP/PKA/CEBP*δ* pathway in TSPCs. Together with BMP-2, IGF-1 can mediate PGE2-induced TSPCs adipogenic differentiation in vitro [34].

#### 5.1.4. Bone Morphogenetic Proteins (BMPs)

BMPs act as differentiation factors and physiological regulators in the homeostasis of different tissues. These proteins have been isolated from bones of various species. BMPs are divided into four subgroups: BMP2/4, BMP5/6/7/8a/8b, BMP9/10, and BMP12/13/14 [58, 59]. The BMPs belong to the TGF-*β* superfamily, which consists of approximately 20 members, but not all of them have been shown to be osteoinductive. Bone morphogenetic proteins are highly expressed in bone and cartilage but not in tendon tissue. They are widely used in cartilage, bone, and specialized tendon-bone junction repair [60]. BMP-2 is a potent osteogenic factor that participates in normal bone healing and ectopic bone formation in soft tissues. Clinical tendinopathy samples have shown that chondro-osteogenic BMPs, including BMP-2, BMP-4, and BMP-7, are expressed. However, healthy control groups do not express BMPs [6]. BMP-2, BMP-4, and BMP-7 were also expressed in the collagenase-induced tendon injury model [8]. These chondro-osteogenic BMPs induce tendon stem cell differentiation into chondrocytes and osteoblasts [26, 35]. In addition to osteogenic and chondrogenic differentiation, Kang et al. found that BMP-2, BMP-4, BMP-6, BMP-7, and BMP-9 induced the adipogenesis of mesenchymal progenitor cells [61]. IGF-1 and BMP-2 together mediate PGE2-induced adipogenic differentiation of TSPCs in an in vitro study [34]. Recent research showed that BMP-2 could promote proteoglycan deposition and induce chondrogenic differentiation of human Achilles TSPCs in vitro [36]. The transplantation of muscle-derived stem cells expressing noggin inhibited BMP-2/4/7-induced ectopic bone formation [62]. In addition, the bone morphogenetic protein receptor-I- (BMPR-I-) specific inhibitor (LDN-193189) can effectively inhibit heterotopic ossification formation in an animal model [63]. Therefore, the chondroosteogenic BMPs have the ability to drive TSPCs into nontenogenic lineages other than tenogenic lineage.

#### 5.1.5. Dexamethasone (Dex)

Glucocorticoids such as Dex have been commonly used to alleviate the inflammation and pain of tendinopathy. However, glucocorticoid-induced tendon rupture is very common in clinical practice. Zhang et al. showed that Dex stimulated cell proliferation at lower concentrations and decreased cell proliferation in a high concentration. Moreover, Dex treatment induced nontenocyte differentiation of hTSPCs in vitro, and implantation of Dex-treated hTSPCs for 3 weeks resulted in the extensive formation of fatty tissues, cartilage-like tissues, and bony tissues in vivo [37]. Chen et al. suggested that Dex inhibits the differentiation of TSPCs to tenocytes by inhibiting the scleraxis gene [38]. These findings indicated that Dex depleted the stem cell pool and led to the formation of nontendinous tissues, which make tendon susceptible to rupture.

### 5.2. Tenogenic Differentiation Factors

#### 5.2.1. Growth Differentiation Factors (GDFs)

GDF-5/BMP-14, GDF-6/BMP-13, and GDF-7/BMP-12 are members of the TGF-*β* superfamily. Wolfman et al. reported that the implantation of GDF-5, GDF-6, and GDF-7 in vivo could induce neotendon formation [64]. Furthermore, GDF-5 and GDF-6 knockout mice displayed thinner tendon tissue and decreased tail tendon collagen production [65–67]. Bolt et al. reported that GDF-5/BMP-14 produced neotenocytes and improved tendon strength [68]. Human MSCs transfected with GDF-5/BMP-14 increased collagen type I, collagen type II, scleraxis, and Runx2 mRNA expression but did not affect osteocalein, tenascin C, or ALP activity [69, 70]. Chai et al. showed that GDF-6 had tenogenic effect on the tenogenic differentiation of BMSCs, and GDF-6 (20 ng/mL) had better tenogenic effect compared to other concentrations [71]. Haddad-Weber et al. found that GDF-6/BMP-13 drove MSCs to tenogenic differentiation [72]. Lee et al. indicated that brief stimulation with BMP-12 in vitro was sufficient to induce BM-MSC differentiation into tenocytes and that this phenotype was sustained in vivo [73]. GDF-7/BMP-12 upregulated decorin and tenomodulin mRNA expression of horse BMSCs in vitro, which further supported their tenogenic differentiation potential [74]. GDF-5 treatment of TSPCs increased the expression of decorin, scleraxis, and collagen type I, whereas adipogenic and chondrogenic markers decreased, suggesting that GDF-5 promoted the transition of TSPCs towards tenocytes [39]. However, the effect of GDF-6/BMP-13 and GDF-7/BMP-12 on TSPCs has not been reported.

#### 5.2.2. Connective Tissue Growth Factor (CTGF)

CTGF is also named CCN2 and was initially identified in the culture supernatant of vascular endothelial cells. Unlike other CCN family members, the CCN2/CTGF gene is conserved among all vertebrates and several invertebrates. Importantly, CCN2/CTGF induces the development and regeneration of mesenchymal tissues, including bone, cartilage, and blood vessels. Furthermore, CTGF can promote fibroblast proliferation and matrix formation in vitro [75]. Chen et al. observed increased CTGF mRNA expression during early tendon healing in a chicken flexor tendon injury model [76]. Würgler-Hauri et al. reported increased CTGF expression during tendon to bone repair [77]. Ni et al. built engineered scaffold-free tendon tissue (ESFTT) via TSPCs, CTGF, and ascorbic acid, and the ESFTT showed significantly increased tenogenic differentiation of TSPCs and decreased osteogenic and chondrogenic differentiation potential [40]. Lee et al. reported that BMSCs treated with CTGF and ascorbic acid induced fibroblastic differentiation but not osteogenic, chondrogenic, or adipogenic differentiation [78]. A recent study reported that CTGF contributed to tendon regeneration by inducing a transient increase in TSPCs and CTGF stimulated proliferation and tenogenic differentiation of TSPCs both in vitro and in vivo [41]. The findings support the use of endogenous stem/progenitor cells as a strategy for tendon regeneration.

#### 5.2.3. Low Oxygen Tension (LOT)

Approximately 1–4% of total nucleated cells in the tendon are TSPCs [10, 11]. Sufficient numbers of healthy TSPCs are essential for transplantation to allow for tendon regeneration and repair. The anatomical site of tendon tissue is relatively oxygen-deficient, which means low oxygen may be necessary for tendon stem cell culture. BMSCs have been reported to maintain higher proliferation, multidifferentiation potential, increased colony formation, and higher cellular metabolism at 2% O_2_ tension [79]. Zhang et al. observed that tenocytes cultured at low O_2_ tension significantly increased their proliferation capacity without affecting their function and phenotype [80]. Lee et al. first described that human TSPCs cultured under 2% O_2_ tension increased cell number by 25%, colony number, and mRNA expression of the tendon-related marker tenomodulin but reduced the osteogenic, adipogenic, and chondrogenic differentiation potentials [42]. In in vitro experiment, TSPCs cultured under 5% O_2_ showed greater cell proliferation and stem cell marker expression than cultured in 20% O_2_; furthermore, when the stem cells were implanted to tendon-derived matrix, more tendon-like structures formed in 5% O_2_ than in 20% O_2_ [43]. Therefore, hypoxia is advantageous for maintaining the stemness of TSPCs in culture and efficient TSPCs expansion in vitro for tendon tissue engineering.

#### 5.2.4. Platelet-Rich Plasma (PRP)

PRP is enriched with critical growth factors and tissue repair pathway mediators. The theoretical basis for the application of PRP in tissue repair stands on certain growth factors and other cytokines in healing various injuries. Ricco et al. suggested that the association between allogeneic adipose tissue-derived mesenchymal stem cells and PRP is a safe and effective strategy for the treatment of superficial digital flexor tendonitis in the horse [81]. Chen et al. reported that adult rat TSPCs cultured with autologous platelet-rich clot releasate (PRCR) induced tenocyte differentiation and suppressed the adipocyte, chondrocyte, and osteocyte lineages in response to 8% mechanical stretching [44]. TSPCs and PRP treatment exerted a synergistic effect on the upregulation of tendon-related gene and protein expression, including collagen type I, scleraxis, and tenascin C, in a collagenase-induced tendinopathy model [46]. Zhang and Wang demonstrated that PRCR dose-dependently promoted tendon stem cell differentiation into active tenocytes and collagen type I, collagen type III, and tenascin C mRNA expression, while decreasing osteogenic (Runx2), chondrogenic (Sox9), and adipogenic (PPAR*γ*) markers [45]. In an in vitro model, PRCR treatment of TSPCs blocked the nontenogenic differentiation when TSPCs were cultured in differentiating media; however, PRCR treatment after pretreatment of TSPCs in nontenogenic media for one week had little effect on any of the three nontenogenic differentiations of TSPCs. That is to say that the injection of PRP in clinics may not be able to effectively reverse the degenerative conditions of late-stage tendinopathy [47]. Nevertheless, because PRP is composed of many components, the role of each factor in TSPCs tenogenic differentiation requires further study.

#### 5.2.5. Biomaterial Scaffold Engineering (BSE)

With a combination of cells, biomaterial scaffolds, and suitable biochemical and physiochemical factors, tissue can be formed to improve or replace biological functions. Tissue engineering is closely associated with applications that repair or replace portions of or whole tissues. Cells within an artificially created support system have also been applied to perform specific biochemical functions under certain mechanical and structural properties. Scaffold elasticity, stiffness, composition, and matrix micro-/nanostructure can modulate cell alignment, migration, proliferation, and differentiation. Pek et al. demonstrated that matrix stiffness affects MSCs phenotypes and differentiation [82]. MSCs differentiated into neural, myogenic, or osteogenic phenotypes depending on whether they were cultured on two-dimensional (2D) substrates of elastic moduli in the lower (0.1–1 kPa), intermediate (8–17 kPa), or higher ranges (34 kPa) [82]. Chen et al. found that overexpression of scleraxis in hESC-MSCs seeded on a knitted silk-collagen sponge scaffold could promote tendon regeneration [83]. Aligned MSCs in a three-dimensional (3D) aligned silk fibroin hybrid scaffold achieved enhanced tenogenesis under mechanical stimulation, as demonstrated by the upregulation in tendon/ligament-related protein expression and production [84]. Yin et al. verified that the aligned nanofiber structure provided an instructive microenvironment for human TSPCs tenogenic differentiation while hindering osteogenic differentiation [19]. TSPCs seeded in a knitted silk-collagen sponge scaffold could differentiate into tenocytes and secrete anti-inflammatory cytokines to enhance rotator cuff tendon regeneration [48]. More recently, engineered acellular extracellular matrix has been used to construct various engineering tissues. Zhang et al. showed that engineered tendon matrix (ETM) in vitro was able to stimulate TSPC proliferation and better preserve the stemness of TSPCs than plastic culture surfaces; moreover, in vivo, implantation of ETM-TSPC composite promoted tendon-like tissue formation [31]. Jiang et al. reported that decellularized fibroblast-derived matrix (dFM) was suitable for growth and tenogenic differentiation of TSPCs in vitro. Neotendon tissue was formed with tendon-specific protein expression when TSPCs were implanted together with dFM [49]. Engineered scaffold-free tendon tissue (ESFTT) is another new biomaterial which is produced via TSPCs by treatment of CTGF and ascorbic acid in vitro. After ESFTT implanted into the nude mouse, neotendon formation could be showed in vivo, and ESFTT could significantly promote tendon healing in a rat patellar tendon window injury model [40].

## 6. New Strategies to Redirect Sufficient Healthy TSPCs into Tenogenic Differentiation

Based on the former hypothesis, erroneous (nontenogenic) TSPC differentiation and aging could account for tendinopathy pathogenesis. Studies have shown that TSPCs are a good alternative cell source for tendon healing. Therefore, the maintenance of a healthy stem cell pool and the redirection of TSPCs to tenogenic differentiation will bring new prospects for the treatment of tendinopathy.

### 6.1. Regulation of Endogenous TSPCs

**Figure 2:**
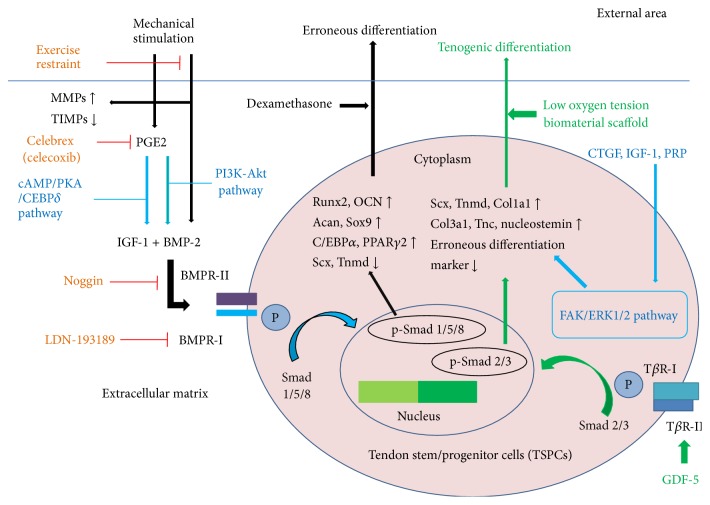
Potential regulations of endogenous TSPCs.

Based on the possible regulatory factors, we propose the following cellular and molecular mechanism of tendinopathy by TSPCs. Mechanical stimulation could increase PGE2 production and disrupt the balance between MMPs and TIMPs [51]. TSPCs then increase BMP-2 expression after mechanical stimulation or through PGE2 induction through the PI3K-Akt signaling pathway [26, 57]. PGE2 increases IGF-1 expression through the cAMP/PKA/CEBP*δ* signaling pathway [34]. BMP-2 could activate Smad 1/5/8 to phosphorylate Smads through BMPR-I and BMPR-II binding at the cell membrane, which could further induce erroneous (nontenogenic) TSPC differentiation by upregulating the corresponding mRNA expression levels (Figure 2).

TSPCs are present at a low ratio, approximately 1–4% of total nucleated cells [10, 11]. Under normal physiological conditions, TSPCs are quiescent, as proliferation and differentiation do not occur. Damage to the ECM can stimulate TSPCs to nontenogenic differentiation. TSPCs isolated from rat tendon tissue could achieve a higher proliferation capacity and produce increased tendon-related collagens under lower (4%) stretching, in contrast to higher (8%) stretching [25]. Increased PGE2 production after mechanical loading, which promotes three-lineage differentiation instead of tenogenic differentiation, is responsible for erroneous tendon stem cell differentiation. Clinical patellar tendinopathy samples showed ectopic BMP-2, BMP-4, and BMP-7 expression and ossification, proteoglycan deposition, and GAG production [6, 35]. TIMPs and aprotinin injected into the target area induce a rebalance of MMPs and TIMPs to prevent collagen degeneration and to create a favorable extracellular matrix environment for TSPC regeneration. Restraining the patients' exercise intensity can promote TSPC tenogenic differentiation. Celebrex (celecoxib), which is a specific COX-2 inhibitor without COX-1 inhibition, reduces PGE2 production and is a possible strategy to prevent erroneous differentiation. Lui and Wong showed that TSPCs isolated from the collagenase-induced tendon injury model were more sensitive to BMP/Smads [85]. Noggin injection might inhibit BMP-induced ectopic bone formation through the BMP/Smad pathway [62]. The BMPR-I-specific inhibitor LDN-193189 might be used to redirect TSPCs to tenogenic differentiation for tendon healing [63]. IGF-1 could promote proliferation and maintenance of TSPC phenotypes with increased decorin and scleraxis expression [39]. GDF-5, GDF-6, GDF-7, CTGF, IGF-1, and autologous PRP are appealing targets to regulate endogenous TSPCs for tenogenic differentiation. The combination of different factors will be important to promote tendon formation, healing, and repair.

### 6.2. Regulation of Exogenous TSPCs

TSPCs have been successfully isolated from human tendon tissue, though the number markedly decreases with aging and degeneration [24]. Hence, the expansion of TSPCs in early passages in vitro is required to obtain a sufficient number of healthy cells, and the rejuvenation of aged TSPCs may provide a possible resolution to improve tendon healing. These cells can be expanded in vitro to more than 10^10^ for tissue repair through various control measures, such as a hypoxic environment (2% O_2_) and implantation in a decellularized tendon matrix. TSPC proliferation capacity significantly increases at low O_2_ tension [42]. The knockout of senescence-associated genes in TSPCs, such as p16^INK4A^, could potentially rescue cellular senescence and regain the natural cellular phenotype. The reinfusion of amplified sufficient healthy stem cells in vitro will become a beneficial approach. Tumor induction by transplanted undifferentiated BMSCs must be taken into consideration [86]. BMSCs have also been reported to induce ectopic bone formation after transplantation, which may be due to changed optimal conditions that drive tenogenic differentiation [87]. These complications will limit their application. The percentage of TSPCs in tendons is at least 3 times higher than MSCs, and complications of the former have not been noted [10]. Collagen production, ultimate stress, and Young's modulus were shown to be significantly increased in the fibrin glue carrier with TSPCs into a rat patellar tendon defect window, and this work implied earlier and better tendon repair [88]. Scleraxis overexpression in hBM–MSCs induced tenomodulin upregulation, which is considered the best-known marker of tenogenic differentiation, and tendon progenitors were successfully converted in vitro [89]. Clinical and animal tendinopathy samples showed altered tendon stem cell fates. BMPs, PGE2, and chondrogenic, osteogenic, and adipogenic phenotypes were observed in the extracellular matrix, which might direct the nontenogenic differentiation of TSPCs. Thus, we suggest the tenogenic differentiation of TSPCs prior to cell transplantation. GDF-5, GDF-6, GDF-7, CTGF, IGF-1, and PRP are promising factors to induce tenogenic differentiation of TSPCs in vitro before local implantation or systemic infusion in vivo. The transduction of tendon-specific genes to TSPCs prior to transplantation is an alternative choice. TSPCs could be cultured with CTGF and ascorbic acid under lower (4%) stretching at lower O_2_ (2%) tension to promote tenogenic differentiation and promote neotendon formation prior to direct delivery to the target site. With tendon stem cell accessory biomaterials, for example, knitted silk-collagen sponge scaffold, silk fibroin hybrid scaffold, aligned PLLA scaffold, and P(LLA-CL)/Col scaffold, relatively better tendon tissue can be achieved before implantation into the wound window under appropriate stimulation. New frontiers will be opened in tendinopathy management through the regulation of exogenous TSPCs.

Although some strategies to redirect sufficient TSPCs into tenogenic differentiation for tendinopathy therapy have been proposed, whether the maintenance of a healthy stem cell pool and the redirection of TSPCs to tenogenic differentiation could work are still unclear. More preclinical studies (including in vitro TSPCs isolation, characterization, expansion, modification and pretreatment, etc.; ex vivo cell delivery safety; in vivo efficiency of the injection of proteins with different functions or TSPCs implantation in animal model of tendinopathy; in vivo malignant cell transformation checking) are required to translate the application of TSPCs for clinical treatment in tendinopathy. At present, there are few clinical trials on the area of TSPCs-based strategies for the treatment of tendinopathy, and we also still lack reliable clinical evidence to evaluate their therapeutic effect. Therefore, further research and exploration of how the various strategies could be used in clinical trials are needed.

## 7. Conclusions

Treatment of tendinopathy still only controls the symptoms. TSPCs are promising candidates for tendinopathy therapy. However, the investigation of these stem-cell-based strategies is limited in the preclinical stage, and the potential role of TSPCs requires further confirmation. In this review, we summarized histopathological changes of tendinopathy, described characteristics of TSPCs, and listed erroneous and tenogenic differentiation factors of TSPCs, which might account for the pathogenesis. The depletion of the stem cell pool and erroneous (nontenogenic) differentiation of TSPCs are a feasible source of tendinopathy pathogenesis. Allogeneic TSPCs could be used, as they are not immunogenic, and surgeons could avoid the difficulties in collecting autologous TSPCs without causing surgical incisions of the donor site. We also discussed new strategies to maintain a healthy stem cell pool and redirect erroneous differentiation to tenogenesis. This information is essential for the future clinical application of TSPCs to treat tendinopathy. An understanding of the mechanisms underlying the aging and erroneous differentiation processes could lead to a major breakthrough in tendinopathy prevention and treatment. These potential strategies will be attractive, promising, and a substantial remedy for defects in the musculoskeletal system.

## Figures and Tables

**Table 1:** Regulatory factors for TSPCs differentiation.

Factors	Cell source	Interventional details	Differentiation	Results	Study type	References
Mechanical stimulation (MS)	New Zealand white rabbits	Cyclic stretching of 4% or 8% at 0.5 Hz for 12 h	4% stretching: tenogenic differentiation 8% stretching: nontenogenic differentiation	Lower stretching (4%) promoted tenogenic differentiation of TSPCs Higher stretching (8%) inhibited tenogenic differentiation of TSPCs	In vitro	Zhang and Wang 2010 [25]

Mechanical stimulation (MS)	Rat	0%, 4%, and 8% stretching at 0.5 Hz for 4 h	Osteogenic differentiation	Repetitive tensile loading increased the expression of BMP-2 and addition of BMP-2 enhanced osteogenic differentiation	In vitro	Rui et al. 2011 [26]

Mechanical stimulation (MS)	Mouse	In vivo: treadmill running In vitro: mechanical stretching (4% and 8%)	Appropriate mechanical loading: tenogenic differentiation Excessive mechanical loading: nontenogenic differentiation	In vivo: moderate running upregulated tenocyte-related genes; intensive running upregulated both tenocyte and nontenocyte-related genes In vitro: low mechanical stretching (4%) increased the expression of only the tenocyte-related genes; high mechanical stretching (8%) increased the expression of both tenocyte and nontenocyte-related genes	In vitro and in vivo	Zhang et al. 2013 [27]

Mechanical stimulation (MS)	Oryctolagus cuniculus	4% dynamic stretching at 0.5 Hz, 2 h per day for a total of 14 days	Tenogenic differentiation	4% dynamic mechanical stimulation increased cell proliferation, tenascin C, decorin, biglycan, and collagen type I and III (tendon-related markers) expression in TSPCs embedded in a P(LLA-CL)/Col scaffold	In vitro and in vivo	Xu et al. 2014 [28]

Mechanical stimulation (MS)	Rat	Uniaxial mechanical tension at 8% elongation (frequency: 1 Hz; 48, 60, or 72 hours)	Osteogenic differentiation	8% uniaxial mechanical stress promoted osteogenic differentiation of rat TSPCs	In vitro	Liu et al. 2015 [29]

Mechanical stimulation (MS)	Mouse	In vivo: treadmill running In vitro: mechanical stretching (4% and 8%)	Moderate exercise: tenogenic differentiation	Moderate mechanical stretching (4%) of aging TSPCs in vitro increased the expression of the stem cell marker and the tenocyte-related genes moderate running of aging mice resulted in the increased proliferation rate of aging TSPCs in culture, decreased lipid deposition, proteoglycan accumulation, and calcification, and increased the expression of nucleostemin	In vitro and in vivo	Zhang and Wang 2015 [30]

Extracellular matrix (ECM)	Mouse	Biglycan and fibromodulin double knockout	Osteogenic differentiation	Expression of scleraxis and of type I collagen decreased, and TSPCs formed bone-like tissue instead of tendon tissue compared to wild-type mice	In vitro and in vivo	Bi et al. 2007 [10]

Extracellular matrix (ECM)	Human	Aligned or randomly oriented poly (l-lactic acid) nanofibers	Aligned nanofibers: tenogenic differentiation randomly oriented nanofibers: osteogenic differentiation	Expression of tendon-specific genes was significantly higher in hTSPCs growing on aligned nanofibers than those on randomly oriented nanofibers; the aligned nanofibers induced the formation of spindle-shaped cells and tendon-like tissue	In vitro and in vivo	Yin et al. 2010 [19]

Extracellular matrix (ECM)	New Zealand white rabbits	Engineered tendon matrix from decellularized tendon tissues	Tenogenic differentiation	Engineered tendon matrix may be used to effectively expand TSPCs in vitro and with TSPCs, to enhance repair of injured tendons in vivo	In vitro and in vivo	Zhang et al. 2011 [31]

Prostaglandin E2 (PGE2)	Human	PGE2 (0, 1, 10, and 100 ng/mL)	Osteogenic differentiation	PGE2 dose-dependently decreased cell proliferation and induced osteogenic differentiation of human TSPCs	In vitro	Zhang and Wang 2012 [32]

Prostaglandin E2 (PGE2)	Human	PGE2 (0, 0.01, 0.1, and 1 ng/mL)	Tenogenic differentiation	Lower PGE2 concentrations (<1 ng/mL) increased cell proliferation and the expression of SSEA-4, Stro-1, Nanog, Oct-4, collagen type I, and tenascin C	In vitro	Zhang and Wang 2014 [33]

Prostaglandin E2 (PGE2)	Rat	PGE2 (0, 10, 50, 100, and 200 ng/mL)	Adipogenic differentiation	IGF-1 and BMP-2 together mediate PGE2-induced adipogenic differentiation of TSPCs in vitro via a CREB- and Smad-dependent mechanism	In vitro	Liu et al. 2014 [34]

Bone morphogenetic proteins (BMPs)	Rat	rhBMP-2 (100 ng/mL)	Osteogenic differentiation	BMP-2 promoted osteogenic differentiation of TSPCs	In vitro	Rui et al. 2011 [26]

Bone morphogenetic proteins (BMPs)	Rat	rhBMP-2 (100 ng/mL)	Nontenogenic differentiation	BMP-2 promoted GAG deposition, aggrecan expression, and enhanced nontenocyte differentiation of TSPCs	In vitro	Rui et al. 2013 [35]

Bone morphogenetic proteins (BMPs)	Human	rhBMP-2 (100 ng/mL)	Chondrogenic differentiation	BMP-2 promoted proteoglycan deposition and induced chondrogenic differentiation of hTSPCs in vitro	In vitro	Rui et al. 2013 [36]

Dexamethasone (Dex)	Human	5, 10, 100, and 1000 nM of Dex solutions	Nontenogenic differentiation	Dex treatment depleted the stem cell pool and led to the formation of nontendinous tissues	In vitro and in vivo	Zhang et al. 2013 [37]

Dexamethasone (Dex)	Rat	1000 nM of Dex solutions	Inhibit tenogenic differentiation	Dex inhibited the differentiation of TSPCs to tenocytes by inhibiting the scleraxis gene	In vitro	Chen et al. 2015 [38]

Growth differentiation factors (GDFs)	Rat	1, 10, or 100 ng/mL of GDF-5	Tenogenic differentiation	GDF-5 treated cells exhibited reduced differentiation along adipogenic and chondrogenic pathways after 28 days, and decorin, scleraxis, and collagen type I expression was increased	In vitro	Holladay et al. 2014 [39]

Connective tissue growth factor (CTGF)	Rat	25 ng/mL of CTGF solution	Tenogenic differentiation	CTGF and ascorbic acid treatment significantly enhanced the tenogenic differentiation of TSPCs and inhibited their osteogenic and chondrogenic differentiation	In vitro and in vivo	Ni et al. 2013 [40]

Connective tissue growth factor (CTGF)	Rat	100 ng/mL CTGF treatment	Tenogenic differentiation	CTGF contributed to tendon regeneration by inducing a transient increase in TSPCs and CTGF stimulated proliferation and tenogenic differentiation of TSPCs	In vitro and in vivo	Lee et al. 2015 [41]

Low oxygen tension (LOT)	Human	2% O_2_ tension	Tenogenic differentiation	TSPCs cultured under 2% O_2_ tension increased cell number, colony number, and mRNA expression of the tendon-related marker but reduced the osteogenic, adipogenic, and chondrogenic differentiation potentials	In vitro	Lee et al. 2012 [42]

Low oxygen tension (LOT)	Human	5% O_2_ tension	Tenogenic differentiation	TSPCs cultured under 5% O_2_ showed greater cell proliferation and stem cell marker expression than cultured in 20% O_2_; when the stem cells were implanted to tendon-derived matrix, more tendon-like structures formed in 5% O_2_ than in 20% O_2_	In vitro and in vivo	Zhang and Wang 2013 [43]

Platelet-rich plasma (PRP)	Rat	2% and 10% PRCR after 8% stretching at 0.5 Hz for 12 h	Tenogenic differentiation	TSPCs cultured with PRCR induced tenocyte differentiation and suppressed the adipocyte, chondrocyte, and osteocyte lineages in response to 8% mechanical stretching	In vitro	Chen et al. 2012 [44]

Platelet-rich plasma (PRP)	New Zealand white rabbits	2% and 10% PRCR	Tenogenic differentiation	PRCR treatment promotes differentiation of TSPCs into active tenocytes exhibiting high proliferation rates and collagen production capability	In vitro	Zhang and Wang 2010 [45]

Platelet-rich plasma (PRP)	Rat	10% PRP	Tenogenic differentiation	TSPCs and PRP treatment exerted a synergistic effect on the upregulation of tendon-related gene and protein expression, including collagen type I, scleraxis, and tenascin C, in a collagenase-induced tendinopathy model	In vitro and in vivo	Chen et al. 2014 [46]

Platelet-rich plasma (PRP)	New Zealand white rabbits	10% PRCR	Tenogenic differentiation	10% PRCR treatment of TSPCs blocked their nontenogenic differentiation	In vitro	Zhang and Wang 2014 [47]

Biomaterial scaffold engineering (BSE)	Human	Aligned nanofiber structure	Tenogenic differentiation	Aligned nanofiber structure provided an instructive microenvironment for hTSPC tenogenic differentiation while hindering osteogenic differentiation	In vitro and in vivo	Yin et al. 2010 [19]

Biomaterial scaffold engineering (BSE)	New Zealand white rabbits	Knitted silk-collagen sponge scaffold	Tenogenic differentiation	Allogenous TSPC-seeded knitted silk-collagen sponge scaffold enhanced the efficacy of rotator cuff tendon regeneration by differentiating into tenocytes	In vitro and in vivo	Shen et al. 2012 [48]

Biomaterial scaffold engineering (BSE)	Rat	Decellularized fibroblast-derived matrix (dFM)	Tenogenic differentiation	Neotendon tissue was formed with tendon-specific protein expression when TSPCs were implanted together with dFM	In vitro and in vivo	Jiang et al. 2014 [49]

Biomaterial scaffold engineering (BSE)	Rat	Engineered scaffold-free tendon tissue (ESFTT)	Tenogenic differentiation	After ESFTT implanted into the nude mouse, neotendon formation could be showed in vivo, and ESFTT could significantly promote tendon healing in a rat patellar tendon window injury model	In vitro and in vivo	Ni et al. 2013 [40]
